# Influence of nursing interventions in improving midwives’ knowledge of misoprostol use in the management of postpartum haemorrhage at selected hospitals in Ondo State, Nigeria

**DOI:** 10.11604/pamj.2021.40.238.18474

**Published:** 2021-12-17

**Authors:** Olufunke Mercy Iwaola, Christiana Olanrewaju Sowunmi, Matthew Idowu Olatubi, Gbemisola Bolanle Ogbeye

**Affiliations:** 1 Faculty of Nursing Sciences, University of Medical Sciences, Ondo, Ondo State, Nigeria,; 2 Department of Nursing, Babcock University, Ilisan, Ogun State, Nigeria,; 3 Department of Nursing, Bowen University, Iwo, Osun State, Nigeria,; 4 Department of Nursing, Federal University, Oye Ekiti, Ekiti State, Nigeria

**Keywords:** Nursing educational intervention, midwives, knowledge, misoprostol, postpartum haemorrhage

## Abstract

**Introduction:**

despite large investments in maternal health services in the world, postpartum hemorrhage (PPH) remains a major cause of maternal mortality. Misoprostol is the most available, accessible, and affordable uterotonic agent in the management of the third stage of labor and has been found to be effective in PPH prevention in low-income countries. This study, therefore, assessed the influence of nursing interventions improving midwives´ awareness of misoprostol use in the management of PPH at selected health facilities.

**Methods:**

we conducted a quasi-experimental study in two secondary health institutions in Ondo State. A total of 68 midwives, who consented to participate, were randomly distributed into experimental and control groups respectively. A questionnaire was administered for obtaining information about participants´ knowledge and use of misoprostol in the management of PPH. Midwives in the intervention group were trained using the adapted Pathfinder International Teaching Package on the use of misoprostol in the management of PPH. Data were analyzed using descriptive and inferential statistics.

**Results:**

in the pre-intervention phase, the mean knowledge score of the control group was 7.55 ± 2.57 while that of the experimental group was 8.89 ± 2.57. There was a significant increase in the number of participants knowing the correct dose of misoprostol for the management of PPH after intervention (27.0% vs 81.1% p=0.01). After intervention, there was a significant increase (p=0.01) in knowledge of misoprostol use in the intervention group compared to the control group (14.73 ± 2.57 vs 8.89 ± 2.57).

**Conclusion:**

misoprostol educational intervention was effective in improving knowledge and use of misoprostol. Hence, continuing educational units in hospitals should include periodic training of midwives on the use of misoprostol in PPH prevention.

## Introduction

Postpartum hemorrhage (PPH) is a global health burden, mostly affecting those in the world´s poorest countries. It can be described as excessive bleeding after the delivery of a fetus. According to the American College of Obstetricians and Gynecologists [1], PPH refers to cumulative blood loss ≥1,000 ml or blood loss accompanied by signs and symptoms of hypovolemia within 24 hours of birth. It could be primary or secondary; primary when it occurs within 24 hours of delivery [2] and secondary when it occurs between 24 hours and 6 weeks postpartum [3]. This implies that any excessive bleeding from the genital tract of a post-natal woman in the first few weeks after delivery is PPH. It accounts for about 35% of maternal mortality all over the world [4]. In Ondo State, the maternal mortality ratio is 208 per 100,000 live births with postpartum hemorrhage (30%) as one of the major causes of maternal mortality [5].

The major risk factor is uterine atony which can be managed using conventional uterotonic drugs, such as oxytocin or misoprostol. Although, oxytocin is the best preference, its use is limited by some factors, such as a lack of access to refrigeration for its storage in low-income settings like Nigeria [6]. Consequently, misoprostol is the most prescribed in low-income settings because of its ease of administration, safety profile, affordability, and ease of storage.

Misoprostol was developed by Searl in 1973 and is a prostaglandin E_1_analogue (cytotec), which can cause powerful contractions of the uterus, even when the uterus is fatigued [7]. Misoprostol has been recommended as a substitute to oxytocin since it could act as an active uterotonic agent. Apart of being cost-effective, it can be taken orally, sublingually, rectally and vaginally. Misoprostol does not require refrigeration and it has a long shelf-life [8]. These attributes make it very important and useful in rural settings where the efficacies of other utero-tonic drugs as well as injection safety are not assured. Also, the skilled birth attendants who are supposed to administer injectable uterotonic drugs are usually not available in many rural settings. In addition, the World Health Organisation (WHO) posits that, misoprostol can be used in situations where the active management of the third stage of labor cannot be practiced. In such situations, it can be given by health personnel who are proficient in its use for PPH management [8]. In 2006, the Federal Ministry of Health in Nigeria approved the use of misoprostol and developed clinical guidelines for its use in the management of PPH [9]. The drug was added to the country´s essential drug list in 2011, showing its significance in the prevention and control of PPH [9]. Despite the discovery and availability of this evidence-based drug, evidence shows that misoprostol has not been effectively used by midwives in the course of managing patients with PPH. This is because maternal indices in Nigeria are still poor [10]. Nigeria as a nation has a high maternal mortality ratio (MMR) with a national figure officially put at 512 maternal deaths/100,000 live births [11]. Maternal mortality accounts for 31% of deaths among women of reproductive age in Nigeria [12].

To promote the use of misoprostol as an effective and easily administered drug for the prevention and management of PPH, there is a need for nurses and midwives to be knowledgeable about its use. This is important because they are the principal perinatal care providers. Adequate training in the form of workshops and in-service training can be used to achieve this leap in knowledge. Koblinsky *et al*. [13] opined that, without adequate training and teaching, nurses and nurse-midwives might not demonstrate adequate competence in the use of misoprostol and other uterotonic agents in the management of PPH. This is important because, misoprostol is the most suitable uterotonic drug in most settings in Nigeria, especially at the primary and secondary levels of health care. It would have been better to carry out this study at both primary and secondary levels of health care. However, most of the primary health care centers are not staffed with nurses and midwives. Hence, this study ought to ascertain whether a nursing intervention would influence the knowledge of midwives in the use of misoprostol in the management of PPH in two selected hospitals in Ondo State. Ondo State was chosen because of the government´s strong political will towards reversing the ugly maternal health indices, having put in place several strategies to achieve a reduction in maternal mortality. This study set out to determine the level of knowledge of midwives about the use of misoprostol in the management of PPH in two selected hospitals in Ondo State. It also measured the influence of a nursing intervention on the knowledge of these midwives.

## Methods

**Study location and design:** the study was carried out at State Specialist Hospital, Akure (SSHA) and State Specialist Hospital, Ondo (SSHO). These two secondary levels of health care were chosen because they are the two biggest hospitals in the state and they have the highest patient/client turnover. While SSHA is in the state capital, hence the largest city in the state, SSHO is in Ondo City, the second-largest city in the state. Two groups of quasi-experimental design was used.

**Sampling:** midwives working in the labor and post-natal wards of the two hospitals were recruited. The selection was randomized into experimental and control groups, using the ballot system. The total sample frame was used because of the small number of midwives in these labor and post-natal wards. A total of 68 midwives participated in the study; 31 in the control group and 37 in the experimental group.

**Study instrument:** a structured questionnaire (adapted from Pathfinder International Teaching Package on the use of misoprostol in the management of PPH) and a teaching guide were the instruments used for data collection. The questionnaire consisted of 25 items, divided into two sections. The first section contained 7 questions that assessed the demographic characteristics of the participants while the second part was an 18-item scale that examined the knowledge of midwives in the use of misoprostol in the management of PPH. The highest score obtainable was “18” while the lowest was “0”. The validity of the questionnaire was established through the face and content validity. Each item on the instrument was examined for content clarity, scope, and relevance to the study. The test re-test method of reliability check was adopted to determine the reliability of the questionnaire. The response of the participants in the setting where the questionnaire was pilot-tested was correlated using Spearman correlation coefficient statistical procedure. The scale yielded a correlation coefficient of 0.94.

**Ethical considerations:** ethical approval to conduct the study was obtained from Babcock University Research Ethical Committee (BUHREC) and Ondo State Health Research Ethics Committee (OSHREC) (AD.4693 Vol 11/42). Permission to conduct the study in each of the hospitals was obtained through the medical directors and the heads of nursing services of each hospital. Participants´ informed consent was sought with an assurance of confidentiality and anonymity. All the information retrieved was treated with the utmost confidentiality and participation in the study was entirely voluntary.

**Study procedure:** the recruitment of participants was done by the researchers and the research assistants. Participants from each setting had the opportunity to choose a convenient date and venue for a meeting within their centers. During the meeting, the purpose, benefits, and stages of the study were explained in detail, and answers were provided to all the questions raised. The schedules of visits were agreed upon by the participants. Pre-intervention data were collected from midwives that volunteered to participate in the study in each of the settings. The date and venue for the training sessions were also agreed upon.

The training was conducted among the experimental group. The training lasted for 180 minutes and was divided into three sessions of 60 minutes each. Each session was subdivided into 45 minutes of teaching and 15 minutes of question and answer. Post-intervention data were collected one week after the intervention. There was no intervention in the control group before the post-intervention data were collected. However, they were trained at the end of the study.

**Data management:** data collated were analyzed using the Statistical Package for Social Sciences (SPSS) version 22. Both descriptive and inferential statistics were used for data analysis. The mean knowledge score of the participants on the use of misoprostol in the management of PPH was determined and compared. Student´s t-test was used to establish the differences between the levels of knowledge of midwives in the experimental and control groups.

## Results

**Socio-demographic characteristics:** the demographic characteristics of the participants in this study, as shown in Table 1, reveals that 42.34 ± 8.39 years was the mean age of the participants in the experimental group while that of the control group was 36.90 ± 7.12 years. Nine point seven percent (9.7%) of the participants in the control group were males while all the participants in the experimental group were females. The mean years of experience as midwives were found to be 11.22 ± 5.24 and 16.62 ± 9.15 in the control and experimental groups respectively.

**Table 1:** socio-demographic characteristics of the participants

		Experimental group (N=37)		Control group (N=31)	
		Frequency	Percentage (%)	Frequency	Percentage (%)
Age in years	20 - 29	1	2.7	5	16.1
	30 - 39	14	37.8	14	45.2
	40 - 49	14	37.8	10	32.3
	50 - 59	8	21.6	2	6.5
Gender	Male	0	0.0	3	9.7
	Female	37	100.0	28	90.3
Marital status	Single	3	8.1	3	9.7
	Married	33	89.2	28	90.3
	Separated/divorced	1	2.7		
Religion	Islam	0	0.0	1	3.2
	Christianity	37	100.0	30	96.8
Professional qualification	RN	36	97.3	25	80.6
	RM	37	100	37	100.0
	RPHN	3	8.1	0	0.0
Academic qualification	Diploma	13	35.1	18	56.1
	BSc/BNSc	24	64.9	13	41.9
Years of experience	1-10	12	32.4	18	58.1
	11 - 20	14	37.8	11	35.5
	21 - 30	8	21.6	1	6.5
	31 - 35	3	8.1	0	0.0

RN: registered nurse; RM: registered midwife; RPHN: registered public health nurse

**Pre-intervention knowledge of the use of misoprostol in the management of PPH:** as shown in Table 2, the experimental group demonstrated a much higher prior knowledge of the use of misoprostol in the management of PPH in all areas tested in comparison with the control group. They had a significantly higher proportion of correct responses (91.9% vs 61.3%, χ^2^= 9.187, p = 0.01) as to whether misoprostol comes in tablet only or not. Also, they had a significantly higher proportion of correct responses (75.7% vs 51.6%, χ^2^= 4.277, p = 0.04) to whether misoprostol is administered rectally. Furthermore, they had significantly higher correct response (89.2% vs 48.4%, χ^2^= 13.526, p = 0.01) to the recommended dose of misoprostol in the management of PPH. The mean pre-intervention knowledge of the use of misoprostol in the management of PPH in the control and experiment groups were found to be 7.55 ± 2.57 and 8.89 ± 2.57 respectively (Table 3). Seventeen (54.9%) of the midwives in the control group had a low knowledge as against 14 (37.8%) in the experimental group. Those that had a high knowledge in the control group were observed to be one (3.2%) compared to three (8.1%) in the experimental group.

**Table 2:** pre-intervention distribution of correct responses on the knowledge of misoprostol in the management of PPH

Knowledge Item	Control Group (n=31)	Experimental Group (n=37)	χ2
	Frequency (%)	Frequency (%)	
Misoprostol is a synthetic prostaglandin analogue	26 (83.9)	36 (97.3)	3.780
Misoprostol comes in tablet form only	19 (61.3)	34 (91.9)	9.187
It comes only in injectable form	20 (64.5)	21 (56.8)	0.515
It is administered rectally	16 (51.6)	28 (75.7)	4.277
It is administered vaginally	25 (80.6)	27 (73.0)	0.552
It is administered sublingually	20 (64.5)	32 (86.5)	4.525
Its recommended dose in the management of PPH is 400mcg	10 (32.3)	10 (27.0)	0.222
Its recommended dose in the management of PPH is 600mcg	15 (48.4)	33 (89.2)	13.526
Its recommended dose in the management of PPH is 800mcg	10(32.3)	15 (40.5)	0.498
Its recommended dose in the management of PPH is 1000mcg	12 (38.7)	10 (27.0)	1.052
Misoprostol works by stimulating the contraction of smooth muscles	30 (96.8)	36 (97.3)	0.016
It stimulates posterior pituitary	9 (29.0)	11 (29.7)	0.004
It stimulates anterior pituitary	13 (41.9)	18 (48.6)	0.306
It closes the cervix, thereby reducing bleeding	7 (22.6)	12 (32.4)	0.813
Misoprostol causes high temperature	18 (58.1)	18 (48.6)	0.600
It causes hypothermia	11 (35.5)	15 (40.5)	0.183
It causes hypotension	10 (32.3)	15 (40.5)	0.498
Misoprostol causes rigor and chills	25 (80.6)	32 (86.5)	0.424

**Table 3:** comparison of existing (pre-intervention) knowledge levels of midwives on the use of misoprostol in the management of PPH in the control and experimental groups

Knowledge item	Pre-intervention (n=37)	Post- intervention (n=37)	χ2	p-value
	Frequency (%)	Frequency (%)		
Misoprostol is a synthetic prostaglandin analogue	36 (97.3)	36 (97.3)	0.000	1.000
Misoprostol comes in tablet form only	34 (91.9)	33 (89.2)	0.158	0.691
It comes only in injectable form	21 (56.8)	30 (81.1)	5.11	0.024
It is administered rectally	28 (75.7)	35 (94.6)	5.232	0.022
It is administered vaginally	27 (73.0)	36 (97.3)	8.649	0.003
It is administered sublingually	32 (86.5)	33 (89.2)	0.126	0.722
The recommended dose in the management of PPH is 400mcg	10 (27.0)	30 (81.1)	21.765	0.001
The recommended dose in the management of PPH is 600mcg	33 (89.2)	31 (83.8)	0.462	0.496
The recommended dose in the management of PPH is 800mcg	15 (40.5)	35 (94.6)	24.667	0.001
The recommended dose in the management of PPH is 1000mcg	10 (27.0)	26 (70.3)	13.848	0.001
Misoprostol works by stimulating the contraction of smooth muscles	36 (97.3)	35 (94.6)	0.347	0.556
It stimulates posterior pituitary	11 (29.7)	22 (59.5)	6.618	0.010
It stimulates anterior pituitary	18 (48.6)	30 (81.1)	8.538	0.003
It closes the cervix, thereby reducing bleeding	12 (32.4)	15 (40.5)	0.525	0.479
Misoprostol causes high temperature	18 (48.6)	34 (91.9)	16.559	0.001
It causes hypothermia	15 (40.5)	24 (64.9)	4.391	0.036
It causes hypotension	15 (40.5)	24 (64.9)	4.391	0.036
Misoprostol causes rigor and chills	32 (86.5)	36 (97.3)	2.902	0.088

**Pre and post-intervention knowledge of the use of misoprostol in the management of PPH:** Table 3 shows the frequency distribution of correct responses with regards to the pre and post-intervention knowledge of the use of misoprostol in the management of PPH in the experimental group. Post-intervention results show that they had a significantly higher proportion of correct responses (81.1% vs 56.8%) to whether misoprostol comes only in injectable form or not (χ^2^= 18.872, p = 0.01). Again, they had a significantly higher proportion of correct responses (94.6% vs 75.7%) to rectal administration of misoprostol (χ^2^= 5.232, p = 0.02). This is also true for the possibility of vaginal administration of misoprostol (97.3% vs 73.0%, χ^2^= 8.649, p = 0.01). In addition, the table shows that a higher proportion of the midwives in the experimental group had a significantly higher proportion of correct responses (81.1% vs 27.0%) on the correct dose of misoprostol in the management of PPH (χ^2^= 21.765, p = 0.01). Most of the midwives at the post-intervention phase had a higher proportion of correct responses (59.5% vs 29.7%) that misoprostol stimulates posterior pituitary (χ^2^= 6.618, p = 0.02). The mean knowledge score of the midwives in the use of misoprostol in the management of PPH increased at the time of post-intervention (8.89 ± 2.57 vs 14.73 ± 2.57) (Table 4). The table further shows that, only three (8.1%) of the midwives had a high knowledge of misoprostol use in the management of PPH at pre-intervention, compared to 29 (78.4%) at post intervention. Paired sample t-test (which compares the mean knowledge of the use of misoprostol in the management of PPH) for both pre and post-intervention for the experimental group shows that participants had a significantly better knowledge in the use of misoprostol in the management of PPH at the time of post-intervention (t = -6.935, p = 0.01).

**Table 4:** pre and post-intervention frequency distribution of correct responses on knowledge of misoprostol in the management of PPH in the experimental group

Knowledge level of misoprostol	Control				Experimental			
	Pre-intervention		Post-intervention		Pre-intervention		Post-intervention	
	F	%	F	%	F	%	F	%
Low	17	54.9	11	35.5	14	37.8	0	0.0
Moderate	13	41.9		58.0	20	54.1	8	21.6
High	1	3.2	18 2	6.5	3	8.1	29	78.4
Total	31	100.0	31	100.0	37	100.0	37	100.0
Mean	7.55 ± 2.57		8.06 ± 2.59		8.89 ± 2.57		14.73 ± 2.57	
					t = -6.935; p = 0.001			

## Discussion

Misoprostol has a range of potential benefits, which include its rapid absorption, stability at optimum temperature, cost-effectiveness, and ease of administration (oral, rectal and sublingual) [14,15]. In the study, 75.7% and 51.6% of the participants in the experimental and control groups respectively believed that misoprostol can only be administered rectally. This shows deficient knowledge on the mode of administration of misoprostol among the midwives in this study. As midwives who were trained in maternal and child health and are actively practicing in maternity units, they are expected to know the different modes of administration of essential drugs like misoprostol in obstetric care. Although more midwives in the study demonstrated some knowledge of vaginal administration of misoprostol in the management of PPH, it is still far below the expectation for midwifery professionals.

Studies have shown that, the oral administration of misoprostol is very active when compared with oxytocin in the management of the third stage of labor [16]. As a result, the World Health Organization endorses the provision and use of misoprostol by health workers for PPH prevention and management in low resource settings [17,18]. It should therefore be of great concern that some midwives in this study are not aware of the oral administration of misoprostol in the active management of the third stage of labor. Adiri and Ejembi [19] opined that the prophylaxis administration of uterotonic drugs like misoprostol is an effective strategy in the active management of the third stage of labor to prevent PPH. It is expedient for midwives who are skilled birth attendants to be knowledgeable about and skilled in the use of misoprostol so as to effectively prevent and manage postpartum hemorrhage. Findings from this study showed that only a few of the midwives in both groups demonstrated adequate knowledge of the use of misoprostol in the management of PPH at pre-intervention stage, despite the fact that they were registered midwives with appropriate educational training and certification. This corroborates the submissions of Okonofua [20] in a study among primary health care workers in Nigeria which revealed that, only a few health workers in their sample have a high level of knowledge about the use of misoprostol for the prevention and treatment of PPH. Also, Oladapo *et al*. [21] in their earlier study, affirmed that there are severe gaps in the knowledge and skills of staff responsible for maternity services in the management of PPH, using misoprostol in the active management of the third stage of labor.

In another related study in Northern Nigeria, though among women in semi-urban communities, Adiri and Ejembi [19] submitted that, the knowledge of the use of misoprostol in the management of PPH was low. The results of our study might be able to explain the results of Adiri and Ejembi´ study. This is because, if the midwives that are expected to educate the women are themselves deficient in knowledge, it is not surprising that, the recipients of care manifest the same ignorance. Earlier, Ejembi and Prata [22] had also documented the poor knowledge of the use of misoprostol in the active management of the third stage of labor among obstetrics workers.

There was no significant difference in the knowledge scores of midwives in the control and experimental groups at the pre-intervention phase (8.89 vs 7.55, p > 0.05). This is similar to the findings of Adiri and Ejembi [19] where it was observed that, the pre-intervention difference in the knowledge scores of the two communities in their study were not statistically significant. It can be said from our study that, for both groups at pre-intervention, midwives demonstrated a low level of knowledge about how misoprostol is used in the management of PPH and its side effects. This implies that despite their educational background as skilled birth attendants, they are unable to give the strong scientific rationale for any intervention they practice in obstetrics management, especially in the case of PPH.

In a similar study carried out in Northern Nigeria, comparing two communities, Adiri and Ejembi [19] discovered that, there was an improvement in the knowledge of the use of misoprostol at the post-intervention phase n in the experimental group. This is similar to what we observed in our study. This further supported the submissions of Koblinsky *et al*. [13] that without adequate training and teaching, nurses and nurse-midwives might not demonstrate an adequate knowledge in the use of misoprostol and other uterotonic agents in the management of PPH. A significant difference was observed in the knowledge scores of the participants in this study on the use of misoprostol in the management of PPH (P < 0.05). Adiri and Ejembi [19] also documented significant statistical differences in pre-intervention and post-intervention knowledge of participants in their similar research.

**Implication for midwifery/nursing practice:** midwives play a significant role in the reduction of maternal mortality in all the three levels of the health care delivery system. They handle most deliveries. It is clear from the result of this study that, the midwives did not demonstrate an adequate knowledge about the use of misoprostol despite being licensed practitioners. Findings from the study also revealed that, without adequate training and re-training, midwives might not demonstrate this important knowledge. It is therefore necessary that regular in-service training and workshops should be organized for midwives in order to keep them abreast of current developments in the management of PPH. This will help to reduce the maternal mortality ratio.

## Conclusion

The knowledge of midwives in the use of misoprostol in the management of PPH was observed to be poor at the pre-intervention phase. However, with adequate nursing intervention, midwives were found to have better knowledge in this area. It is, therefore, important that efforts should be directed at the regular training of midwives in the use of misoprostol in the management of PPH.

**Recommendations:** based on the findings of this study, the following recommendations are proposed: 1) Up-to-date training on the use of misoprostol in the management of PPH should be organized for midwives to keep them abreast of current practice; 2) the Nursing and Midwifery Council of Nigeria should revise the curriculum for the Schools of Nursing and Midwifery to include contents on the use of misoprostol in the management of PPH.

### What is known about this topic

- *Postpartum hemorrhage is a great burden in many developing nations of the world, including Nigeria and the use of uterotonic agents had been found to the effective in averting PPH;*
- *Among the various uterotonic agents available, misoprostol had been found to be the most affordable and usable drug in low-income settings*.

### What this study adds

- *The study helps in determining that the baseline knowledge of midwives about the use of misoprostol is low;*
- *It provides educational intervention on the use of misoprostol in the management of PPH among the participants;*
- *The study reveals that, the midwife-led misoprostol educational intervention is effective in improving the knowledge and use of misoprostol among the study population*.
